# Face Masks Bolsters the Characteristics From Looking at a Face Even When Facial Expressions Are Impaired

**DOI:** 10.3389/fpsyg.2021.704916

**Published:** 2021-12-10

**Authors:** Wee Kiat Lau

**Affiliations:** Department of General Psychology, Ulm University, Ulm, Germany

**Keywords:** face masks, emotion recognition, traits, facial expressions, face

## Abstract

Face masks impact social interactions because emotion recognition is difficult due to face occlusion. However, is this enough to conclude that face masks negatively impact social interactions? We investigated the impact of face masks on invariant characteristics (sex, age), trait-like characteristics (trustworthiness, attractiveness, and approachability), and emotional expressions (happiness and excitability). Participants completed an online survey and rated masked and no-masked faces. The same face remained masked or no-masked throughout the survey. Results revealed that, when compared to no-masked faces, masked happy faces appeared less happy. Face masks did not negatively impact the ratings of other characteristics. Participants were better at judging the sex of masked faces. Masked faces also appeared younger, more trustworthy, more attractive, and more approachable. Therefore, face masks did not always result in unfavorable ratings. An additional *post hoc* modeling revealed that trustworthiness and attractiveness ratings for masked faces predicted the same trait ratings for no-masked faces. However, approachability ratings for no-masked faces predicted the same trait ratings for masked faces. This hinted that information from masked/no-masked faces, such as from the eye and eye region, could aid in the understanding of others during social interaction. Future directions were proposed to expand the research.

## Introduction

The face communicates several characteristics about a person. These characteristics can be invariant and stable across time, such as the sex and age (Brown and Perrett, 1993; Nkengne et al., 2008). Characteristics can convey trait-like information like attractiveness, trustworthiness, and approachability (Perrett et al., 1999; Scheib et al., 1999; Russell, 2003; Todorov et al., 2008; Vernon et al., 2014; Linke et al., 2016). In some cases, the sex and age of a face also influence how attractive the face looks. Explicitly, feminine and younger faces appear more attractive (Korthase and Trenholme, 1982; Rhodes et al., 2000). Facial expressions can also communicate certain characteristics about a person. For instance, a smiling face often appears more attractive and pleasant (Otta et al., 1996), but an angry face is less approachable (Willis et al., 2011). Thus, we extract, from the face, invariant characteristics, trait-like characteristics, and depend on emotion expressions to better understand a person.

There are often situations where the face is partially occluded due to apparel like scarves, religious coverings, and more recently, face masks (Kret and De Gelder, 2012; Kret and Fischer, 2018; Spinelli and Pellino, 2020). Face masks occlude half the face. This limits several facial features such as the nose and mouth which facilitate social interaction (Seamon et al., 1978; Calvo and Fernández-Martín, 2013; Calvo et al., 2014). Despite having access to only the eyes and eye regions, we do not experience a gross handicap in understanding others.

### The Negative Impact of Face Masks in Social Interactions

Studies investigating the impact of face masks on social interactions have so far implicated negative connotations to the face masks. Specifically, face masks impede one’s ability to recognize discrete facial expressions like happy, sad, and disgust accurately (Carbon, 2020). Often, we become confused by some of the expressions. Other studies show that face masks increase the difficulty at recognizing the identity of the mask wearer (Freud et al., 2020). Interestingly, there are contrary evidence which negate the negative implications of face masks. For instance, children’s ability to recognize facial expressions appear to be spared from the influence of face masks (Ruba and Pollak, 2020).

Negative connotations between face masks and social interactions are often drawn prematurely. An argument about the negative impact of face masks is that certain characteristics about a person can be difficult to interpret due to the mask. This is because facial features communicating such information are occluded by the masks. However, recent literature has shown that face masks may influence social interactions in a complex manner that is not entirely negative. Calbi et al. (2021) demonstrated that a person’s decision to socially distance themselves from others could be dependent on their gender, the type of face masks, and the facial expression of the mask wearer. Males were likely to distance themselves from faces wearing non-protective face masks (i.e., scarfs) and emoting angry expressions. Females, however, distanced themselves regardless of the type of face masks, if the facial expressions were aversive. Hence, it is premature to assume a negative relationship between face masks and social interaction since this relationship is more complicated.

### Do Face Masks Frustrate Social Interactions?

The eyes and eye regions are equally important as they convey information during social interaction. We depend on the eyes to identify certain emotions such as fear, anger, and sadness (Dadds et al., 2006; Wegrzyn et al., 2017). The eyes and the eye region also provide important cues for recognizing faces (Vinette et al., 2004; Royer et al., 2018). For instance, faces are more effectively recognized by looking slightly below the eyes (Peterson and Eckstein, 2012). Therefore, our ability to communicate effectively in social interactions does not involve only regions occluded by face masks, but also the eyes and eye regions.

For the following study, we were interested in the following question: what is communicated from the non-occluded regions (i.e., the eyes and eye regions) that aid social interaction. We compared the evaluation of various characteristics between masked and no-masked faces to understand which characteristics could be inferred from the eyes and eye region. We were interested in invariant characteristics (sex, age) and trait-like characteristics (trustworthiness, attractiveness, approachability) of neutral faces. In a meta-analysis, happiness and excitement were the top two most recognized facial expressions for literate populations (Russell, 1994). Therefore, we evaluated the impact of face masks on perceived excitability and happiness in happy, neutral, and sad faces.

We hypothesized that face masks impeded the ability to infer characteristics communicated mainly by the regions occluded by masks (i.e., the mouth and nose regions). Therefore, ratings for masked faces would be less intense than ratings for no-masked faces. For example, we expected that happiness ratings would decrease for masked faces in contrast to no-masked ones. Since excitement is highly similar to happiness (Russell and Bullock, 1985), face masks would also impede one’s ability to infer excitability. Therefore, we expected excitability ratings to decrease for masked faces in comparison to no-masked ones. A lack of differences between masked and no-masked faces meant that the characteristics were not communicated by areas occluded by the face masks. Subsequently, the results would suggest that the non-occluded facial areas, i.e., the eyes and eye region, were involved in conveying these characteristics.

As discussed previously, trait-like characteristics can be influenced by invariant characteristics and the emotional expressions of a face. However, do face masks affect the evaluation of trait-like characteristics? We examined if the evaluation of trait-like characteristics was dependent on face masks using modeling and model comparisons. First, we derived the optimal (i.e., best) models by fitting invariant characteristics and emotional expression ratings to predict trait-like characteristics. This produced two optimal models, one for masked data and one for no-masked data. Then, we used the optimal model from each trait-like characteristic to predict the alternative data; the optimal model from masked data was fitted onto no-masked data to evaluate fit compatibility, and vice versa. When masked data predicted no-masked data, this indicated that the model components for masked data could be the same ones used for evaluating no-masked data. This would be confirmed if the optimal model for no-masked data also successfully predicted masked data using the same components. Subsequently, we would conclude that face masks did not influence the evaluation of the specific trait-like characteristic.

## Materials and Methods

### Participants

Participants were recruited in accordance with the declaration of Helsinki. 1077 participants completed the online survey between November 19, 2020 and December 18, 2020. There were two survey versions, Version A (*N* = 539, 48%) and Version B (*N* = 538, 52%). Participants were randomly assigned to complete only one version. In general, female participants (*N* = 937, 87%) constituted the majority as compared to male (*N* = 132, 12.3%) and diverse (*N* = 8, 7%) participants. The sample was also predominantly young adults between ages 20–29 years old (*N* = 597, 55.4%).

### Stimuli

**FIGURE 1:**
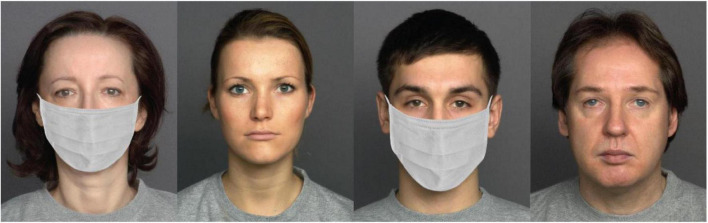
Sample neutral faces used in the survey. Eight faces were used in the experiment. Four of the faces were shown here. There was a no-masked and a masked version for each face.

Face stimuli were adapted from the FACES database (Ebner et al., 2010). The advantage of this database was that all faces were validated. Faces from this database were colored images, varying in age and gender. All faces were of Caucasian origins. We used eight faces (four females, four males) for the survey. These eight faces were also selected based on the face model’s age (four young, four old). Each face exhibited happy, neutral, and sad facial expressions. We added face masks to all faces using Photoshop. Figure 1 illustrates some faces with neutral expressions used in the survey.

Faces presented to each participant had either a mask or were unmasked. From here-on, we refer to the faces as masked and no-masked faces. The study consisted of two versions, Version A and Version B, that establishes which images were masked faces. Half of the faces in each version were masked faces. Masked faces in version A were no-masked faces in Version B. There were two blocks per version. The blocks indicated which facial expressions were displayed during the study. Participants saw only neutral faces in Block 1. In Block 2, participants saw happy, neutral, and sad faces. The survey was administered online using EFS Survey by Questback GmbH (2021).

### Design

The survey was administered either in the German (*N* = 1070, 99.4%) or in the English (*N* = 7, 0.6%) language. There were two versions of the survey. In Version A, two male and two female faces were masked and the remaining four were no-masked faces. We also included an old and a young face for each of the face’s sex. Therefore, there were always two masked old faces (one male and one female) and two masked young faces (one male and one female). In Version B, this mapping was reversed so that masked faces in Version A did not have masks in Version B, and vice versa.

The survey consisted of two sections. Each section measured different characteristics. The first section measured the invariant characteristics (sex, age) and trait-like characteristics (trustworthiness, attractiveness, approachability) in neutral face stimuli. The second section measured the emotional expressions of excitability and happiness of the same face presented with either a happy, neutral, or sad expression. Participants gave their responses by clicking on 10-point Likert-scales for each characteristic. The end poles of each characteristics were label “very …” or “not at all ….” For instance, participants rated whether the current face was “most trustworthy” or “not at all trustworthy.” We then prompted participants for their age, gender, and highest educational qualification. Participants always began with the first section before advancing to the second section.

We now elaborate on some terms used in the writing to avoid confusion. Participants rated the invariant characteristics of sex and the age for each face. Therefore, our dependent variables (DV) for these two characteristics were termed SEX_DV_ and AGE_DV_. Throughout the survey, we presented male and female faces. For each of the face model’s sex, we also showed young and old faces. To prevent any confusion, the terms SEX_IV_ and AGE_IV_ indicated female/male face models and young/old face models, respectively.

Faces in each section were randomly presented. In the first section, neutral faces were comprised of 4 SEX_IV_ (2 females, 2 males) × 2 Mask (masked, no-masked) = 8 faces. Masked faces in Version A were no-masked ones in Version B, vice versa. Participants evaluated five characteristics of all faces presented: SEX_DV_, AGE_DV_, trustworthiness, attractiveness, and approachability. In the second section, there were 4 SEX_IV_ (2 females, 2 males) × 2 Masks (masked, no-masked) × 3 Expression (happy, neutral, or sad) = 24 faces. Half of the faces were masked. Participants first evaluated the excitability of all the faces before rating the happiness. Masked/no-masked faces in each version remained masked/no-masked throughout the survey, regardless of the expressions. Participants never saw the same face with and without masks.

### Procedure

The online survey was administered under no supervision from a research assistant. Participants gained access to the survey through a link via the student mailing lists at Ulm University or via social media (i.e., Facebook and Instagram). Participants were also encouraged to share the survey link on their social media accounts. The language selection screen was first presented after clicking the link. Participants picked either the German or the English language to complete the survey. Thereafter, the informed consent was presented. After providing consent, participants saw the instructions.

Participants were instructed to rate the *person* based on invariant and trait-like characteristics or emotional expressions. They were also told that there were no right or wrong responses. Participants made a key press to acknowledge the instructions and to proceed with a practice trial. In the practice trial, a young female face was shown, and participants judged how attractive the face looked. Participants responded by clicking on the 10-point Likert scale or by sliding a button along the scale. The scale ranged from “not at all attractive” to “very attractive.” The face shown in the practice trial never appeared in the rest of the survey. Responses for the practice trial were also excluded from subsequent analyses. The two sections of the survey ensued.

Participants rated invariant and trait-like characteristics in the first section, and emotional expressions of excitability, then happiness, in the second section. Each characteristic was preceded by instructions describing the scale and the task. For example, “Please rate the person’s trustworthiness. You can choose a point on the scale between *not at all trustworthy* and *very trustworthy.*” In the second section, we provided several adjectives to describe excitability and happiness. These adjectives were derived from previous literature (Mehrabian and Russell, 1974). The adjectives for “not at all excited” were relaxed, calm, sluggish, clumsy, sleepy, and rested. The adjectives for “very excited” were stimulated, excited, turbulent, nervous, awake, and restless. The adjectives for “not at all happy” were worried, annoyed, dissatisfied, moody, sad, desperate, and bored. The adjectives for “very happy” were happy, content, satisfied, comfortable, hopeful, and relaxed. For each emotional expression, participants were tasked to complete a comprehension check by picking an adjective from a list of four which described the expression. Participants who failed the comprehension checks were excluded from the analyses.

Throughout the survey, participants clicked on the “next” button positioned at the bottom right corner of the survey to advance to the next page. On each page, we presented only one face and one scale. The scale was positioned directly below the face with the following prompt: “This person appears to me ….” The total duration of the survey lasted approximately 10–15 min.

### Analysis

All analyses were conducted in SPSS and AMOS (IBM SPSS Statistics for Windows, Version 26.0). The DVs for invariant characteristics were: SEX_DV_ and AGE_DV_. The DVs for trait-like characteristics were: Trustworthiness, Attractiveness, and Approachability. The DVs for emotional expression were: Excitability and Happiness. With respect to our main hypotheses, the IV for invariant and trait-like characteristics was mask (masked and no-masked faces) and the IVs for emotional expressions were mask (masked and no-masked) and facial expressions (happy, neutral, and sad).

Data was excluded from the analyses. Participants who failed either the excitability or happiness comprehension checks, or both, were excluded from the analyses. From this criteria, we discarded 87 (8.08%) responses from the dataset. The final sample was comprised of 990 participants, where 489 (49.4%) participants completed Version A and 501 (50.6%) participants completed Version B of the survey.

The goal of the survey was to evaluate if face masks negatively affected one’s ability to identify characteristics when looking at a face. We assessed the ratings between masked and no-masked data using paired samples *t*-tests. This analysis was performed individually per invariant and trait-like characteristics. Multiple comparisons were accounted using Bonferroni-corrected α = 0.05 ÷ 5 = 0.01. Thus, the comparisons were significantly different if *p* < 0.01. The variable SEX_DV_ was recoded. Participants rated SEX_DV_ along a 10-point scale from “very male” to “very female.” This was recoded to indicate the proportion correct for SEX_IV_ (i.e., the correct face model’s sex). Higher SEX_DV_ reflected greater accuracy.

The hypothesis for emotional expressions of excitability and happiness was tested with a 2 × 3 repeated measures ANOVA. The within-subjects factors were mask and facial expressions, and the interaction between the two factors was estimated. We also evaluated if the sphericity assumption was violated using Mauchly’s W for facial expressions and the interaction effect. Sphericity test was not conducted for the two-level factor, mask, as it would be meaningless (Mauchly, 1940). Greenhouse-Geisser correction was used for comparisons which violated the sphericity assumption (Greenhouse and Geisser, 1959). *Post hoc* analysis for significant main effects and interactions were examined with *t*-tests. Multiple comparisons for the *post hoc* tests were accounted for using Bonferroni-corrected α = 0.05 ÷ 3 = 0.017.

We conducted *post hoc* modeling to investigate if invariant characteristics and emotional expressions influenced trait-like characteristics. This was performed twice, once for masked faces, and once for no-masked faces. This analysis involved multiple steps: correlation analysis, stepwise regression, path analysis, and model comparisons. We first calculated the mean scores for each DV. Ratings of the DVs across all faces were averaged from each participant. We then correlated the DVs using the averaged values to obtain an overview of the relationships between DVs. The correlation matrix helped us to justify if the subsequent stepwise regression analysis correctly excluded highly correlated predictors.

The role of the stepwise regression was to determine the optimal models for predicting Trustworthiness, Attractiveness, and Approachability. We estimated these variables separately to avoid collinearity issues since these variables were moderately correlated with each other (see Figure 5). The stepwise regression was ran separately for masked and no-masked faces. Predictors which were added into the first step of the regression were SEX_DV,_ AGE_DV_, Excitability (Happy, Neutral, Sad faces) and Happiness (Happy, Neutral, Sad faces). The optimal model from the regression quantified the contributions of invariant characteristics and/or emotional expressions on each trait-like characteristic for masked and no-masked faces, respectively. Details concerning the stepwise regression were reported in the Supplementary Appendix.

We then conducted path analysis on the optimal models using the AMOS software to evaluate if the optimal model of one data could predict alternate data. If the optimal model of one data predicted the alternate data, it would indicate that the trait-like characteristic was communicated from the face regardless of the occlusion by face masks. Subsequently, this hinted that the uncovered regions of the face communicated information related to the trait-like characteristic. This analysis was achieved by fitting the optimal models of half the data (e.g., masked data) onto the other half (e.g., no-masked data), vice versa. For example, we conducted path analysis on the optimal model which predicted trustworthiness for masked faces. Then, we fitted this model on data from no-masked faces. We evaluated how well the optimal model fitted the alternate data using recommended model fit parameters by Schreiber et al. (2006): ratio of χ^2^ ÷ *df* ≤ 2, normed fit Index (NFI) ≥ 0.95, and root mean square error for approximation (RMSEA) < 0.06. We also assessed the Akaike information criterion (AIC) value (Akaike, 1973), since a low AIC value represented good model fits. In all our analyses, the significance was determined at 95% confidence interval (*p* < 0.05).

## Results

### Invariant Characteristics

**FIGURE 2:**
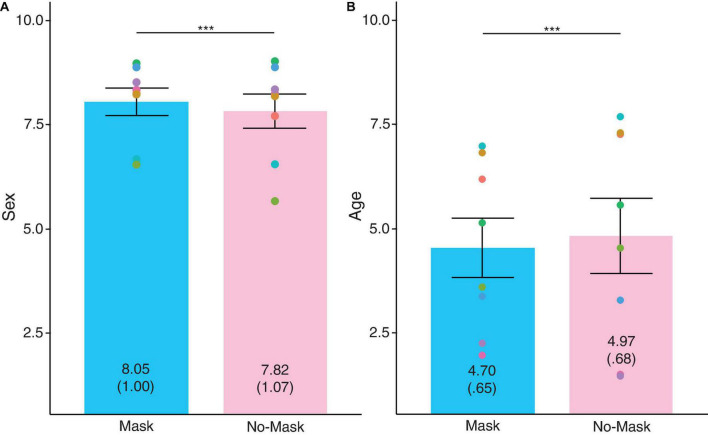
Ratings for invariant characteristics of **(A)** SEX_DV_ and **(B)** AGE_DV_. Individual points represent averaged rating for each face. The values depicted in each bar refer to the mean ratings and the standard deviation is in parenthesis. Error bars indicate standard error means (SEM). Higher values indicate more accurate in **(A)** and older in **(B)**. ***p > 0.001, **p > 0.01, *p > 0.05.

This survey investigated whether the characteristics drawn from a face were negatively affected because of face masks. Ratings between masked and no-masked faces were compared. For invariant characteristics (Figure 2), participants were significantly more accurate at rating the SEX_DV_ of masked faces than no-masked ones, *t*(989) = 7.35, *SD* = 0.98, *p* < 0.001, partial η^2^ = 0.05, Bonferroni-corrected α = 0.01. However, the AGE_DV_ for masked faces appeared younger than no-masked faces, *t*(989) = −11.27, *SD* = 0.76, *p* < 0.001, partial η^2^ = 0.11, Bonferroni-corrected α = 0.01. Therefore, face masks affected the ratings for invariant characteristics. However, the impact was not always negative. When compared to no-masked faces, participants were better at judging the SEX_DV_ of masked faces Masked faces also appeared more youthful.

### Trait-Like Characteristics

**FIGURE 3:**
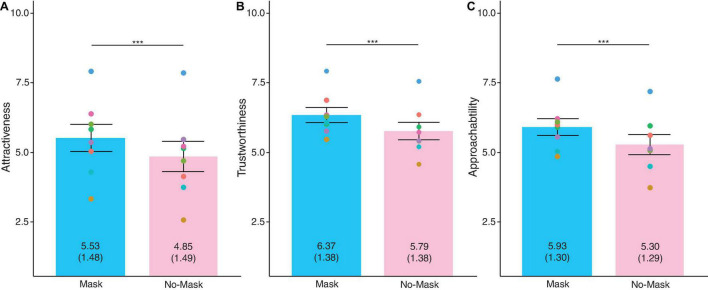
Ratings for trait-like characteristics of **(A)** Attractiveness, **(B)** Trustworthiness, and **(C)** Approachability. Individual points represent averaged rating for each face. The values depicted in each bar are the mean ratings and the standard deviation is in parenthesis. Error bars indicate standard error means (SEM). Higher ratings indicate greater agreement to the perceived traits. ***p > 0.001, **p > 0.01, *p > 0.05.

Trait-like characteristics differed statistically between masked and no-masked faces (Figure 3). Masked faces looked more attractive than no-masked faces, *t*(989) = 12.50, *SD* = 1.71, *p* < 0.001, partial η^2^ = 0.14, Bonferroni-corrected α = 0.01. Masked faces appeared more trustworthy than no-masked versions, *t*(989) = 13.08, *SD* = 1.39, *p* < 0.001, partial η^2^ = 0.15, Bonferroni-corrected α = 0.01. Masked faces were also more approachable than no-masked ones, *t*(989) = 13.00, *SD* = 1.54, *p* < 0.001, partial η^2^ = 0.15, Bonferroni-corrected α = 0.01. From these results, we found that face masks impacted the ratings of trait-like characteristics. Participants attributed higher ratings to the all trait-like characteristics for masked faces.

### Emotional Expressions

#### Excitability

**FIGURE 4:**
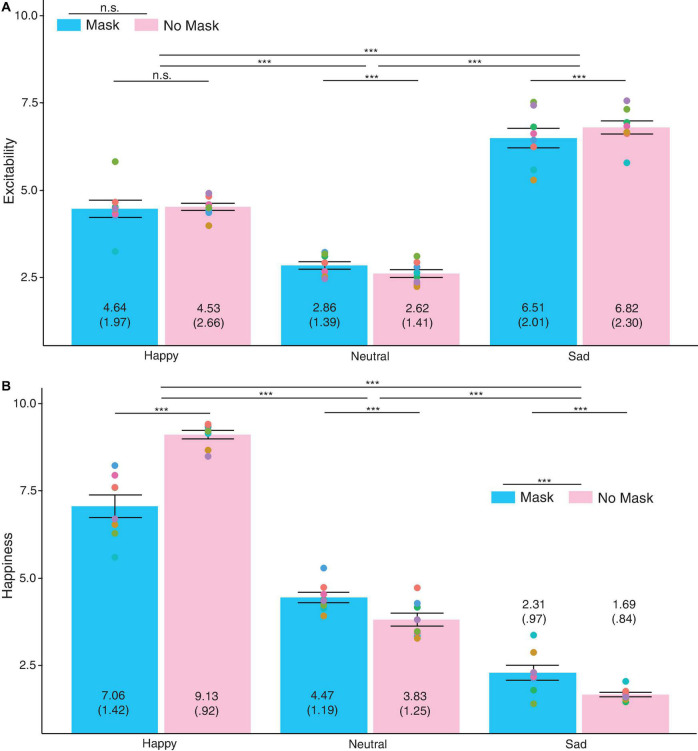
Ratings for emotional expressions of **(A)** Excitability and **(B)** Happiness across happy, neutral, and sad faces. Light blue bars represent masked faces and light pink bars represent no-masked faces. Individual points are the averaged rating for each face. The values depicted in each bar are the mean ratings and the standard deviation is in parenthesis. Error bars indicate standard error means (SEM). Higher ratings indicate greater agreement to the perceived emotional expressions. ***p > 0.001, **p > 0.01, *p > 0.05.

There was no significant main effect of face masks on excitability ratings, F(1, 989) = 2.18, *p* = 0.14, partial η^2^ = 0.002 (Figure 4A). The factor, facial expressions, violated the sphericity assumption, Mauchly’s W = 0.87, *p* < 0.001. Greenhouse-Geisser correction was used. The ratings across the three facial expressions were significantly different, F(1.78, 1757.81) = 1117.00, *p* < 0.001, partial η^2^ = 0.76. A *post hoc* analysis was conducted to identify which facial expressions contributed to the statistical differences. The analysis showed that sad (M = 6.66, *SD* = 2.04) expressions looked the most excitable when compared to happy (M = 4.50, *SD* = 0.2.14), *t*(989) = 22.56, *SD* = 3.02, *p* < 0.001, Bonferroni-corrected α = 0.017, and when compared to neutral (M = 2.74, *SD* = 1.29) expressions, *t*(989) = 55.08, *SD* = 2.24, *p* < 0.001, Bonferroni-corrected α = 0.017. Happy expressions also seemed more excitable than neutral expressions, *t*(989) = 21.88, *SD* = 2.52, *p* < 0.001, Bonferroni-corrected α = 0.017. The interaction between mask and facial expressions violated the sphericity assumption, Mauchly’s W = 0.92, *p* < 0.001. Greenhouse-Geisser correction was used. The interaction between mask and facial expressions was significant, F(1.86, 1834.77) = 35.79, *p* < 0.001, partial η^2^ = 0.04. From Figure 4A, excitability ratings for different facial expressions changed between masked and no-masked faces. However, the changes were not uniformed. Excitability ratings did not differ between masked happy (M = 4.46, *SD* = 1.97) and no-masked happy (M = 4.53, *SD* = 2.66) faces, *t*(989) = −1.02, *SD* = 1.90, *p* = 0.31, Bonferroni-corrected α = 0.017. Excitability ratings differed between masked neutral (M = 2.86, *SD* = 1.39) and no-masked neutral (M = 2.62, *SD* = 1.41) faces, *t*(989) = 6.79, *SD* = 1.11, *p* < 0.001, Bonferroni-corrected α = 0.017. Excitability ratings also differed between masked sad (M = 6.51, *SD* = 2.01) and no-masked sad (M = 6.82, *SD* = 2.30) faces, *t*(989) = −6.97, *SD* = 1.40, *p* < 0.001, Bonferroni-corrected α = 0.017.

**FIGURE 5:**
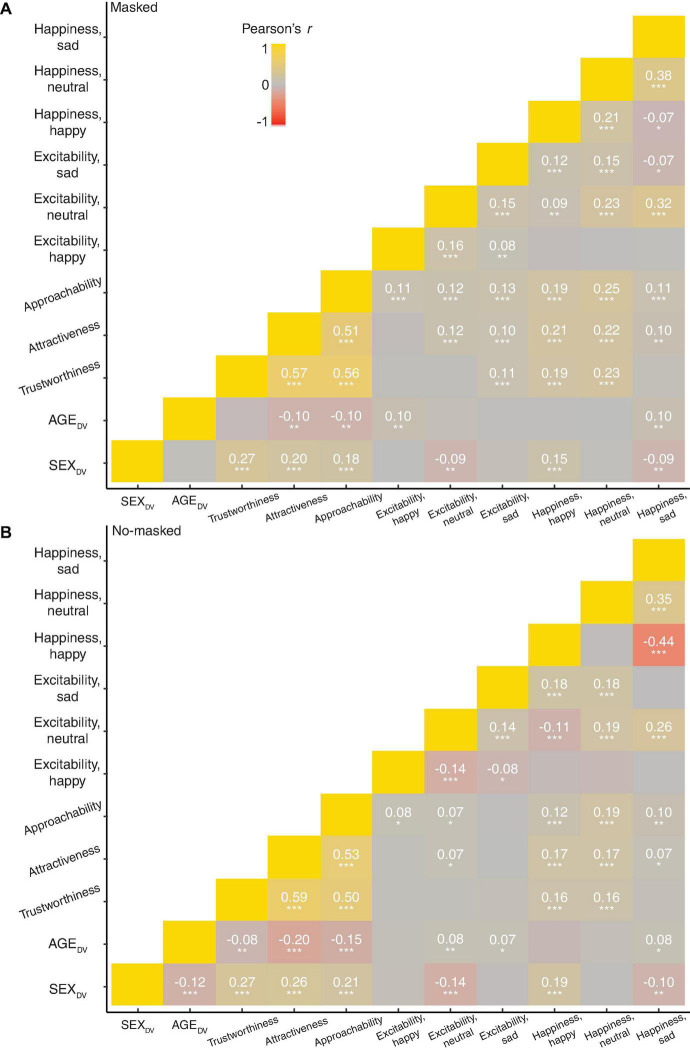
Correlations between dependent variables for **(A)** masked and **(B)** no-masked faces. Color indicates correlation coefficient; Pearson’s *r*. Non-significant coefficients were excluded to reduce visual clutter. ***p ≤ 0.001, **p ≤ 0.01, *p ≤ 0.05.

#### Happiness

There was a significant main effect of mask and happiness ratings, F(1, 989) = 155.99, *p* < 0.001, partial η^2^ = 0.14 (Figure 4B). Masked faces (M = 4.62, *SD* = 0.80) received significantly lower ratings than no-masked (M = 4.88, *SD* = 0.59) faces. The factor, facial expressions, violated the sphericity assumption, Mauchly’s W = 0.91, *p* < 0.001. Greenhouse-Geisser correction was used. Participants rated each facial expression differently, F(1.83, 1811.02) = 11007.99, *p* < 0.001, partial η^2^ = 0.95. *Post hoc t*-tests revealed that happy (M = 8.10, *SD* = 0.96) expressions were rated happiest as compared to neutral (M = 4.15, *SD* = 1.13), *t*(989) = 89.79, *SD* = 1.38, *p* < 0.001, Bonferroni-corrected α = 0.017, and to sad (M = 2.00, *SD* = 0.81) expressions, *t*(989) = 134.17, *SD* = 1.43, *p* < 0.001, Bonferroni-corrected α = 0.017. Neutral expressions were also rated happier than sad expressions, *t*(989) = 61.71, *SD* = 1.10, *p* < 0.001, Bonferroni-corrected α = 0.017. This indicated that the participants attributed the emotional expressions correctly. The interaction factor between masks and facial expressions violated the sphericity assumption, Mauchly’s W = 0.83, *p* < 0.001. Greenhouse-Geisser correction was used. There was a significant interaction effect between mask and facial expressions on happiness ratings, F(1.70, 1684.42) = 2143.76, *p* < 0.001, partial η^2^ = 0.68. Happiness ratings differed significantly between masked happy (M = 7.06, *SD* = 1.43) and no-masked happy (M = 9.13, *SD* = 0.92) faces, *t*(989) = −45.48, *SD* = 1.43, *p* < 0.001, Bonferroni-corrected α = 0.017. Happiness ratings differed significantly between masked neutral (M = 4.47, *SD* = 1.19) and no-masked neutral (M = 3.83, *SD* = 1.25) faces, *t*(989) = 21.28, *SD* = 0.94, *p* < 0.001, Bonferroni-corrected α = 0.017. Happiness ratings differed significantly between masked sad (M = 2.31, *SD* = 0.97) and no-masked sad (M = 1.69, *SD* = 0.84) faces, *t*(989) = 24.00, *SD* = 0.82, *p* < 0.001, Bonferroni-corrected α = 0.017.

The results showed that the effects of face masks on excitability ratings was inconsistent. However, face masks affected happiness ratings across all facial expressions. Participants perceived a drop in happiness for masked faces. Participants also did not misattribute the expressions.

### Correlational Analysis

One may extract information regarding trait-like characteristics of another using invariant characteristics, excitability, and happiness when seeing the full face. However, this ability could be reduced when half the face is covered. We conducted a *post hoc* analysis to evaluate this. We fitted regression models to predict trait-like characteristics (Trustworthiness, Attractiveness, and Approachability) using predictors from invariant characteristics (SEX_DV_, AGE_DV_) and emotional expressions (Excitability, Happiness). In the following sections, we presented systematically the results of our analysis.

We first correlated all the DVs for masked (Figure 5A) and no-masked faces (Figure 5B). Trait-like characteristics were highly correlated with other trait-like characteristics (0.51 < *r* < 0.59, *p* < 0.001), regardless of masked or no-masked faces. However, these characteristics were either uncorrelated to or lowly correlated to invariant characteristics (SEX_DV_, AGE_DV_) and emotional expressions (Excitability, Happiness). Therefore, this suggested that the subsequent regression models should estimate each trait-like characteristics separately, as these variables would show high multi-collinearity when estimated together in the same model. Additionally, there would be no multi-collinearity issues when predicting trait-like characteristics using invariant characteristics and emotional expressions due to the low correlations.

### Path Analysis

Optimal models for each trait-like characteristics were analyzed separately for masked and no-masked data. Models were then fitted to the alternate data (e.g., the optimal model for masked data of Trustworthiness was fitted onto no-masked data of Trustworthiness).

#### Trustworthiness

**FIGURE 6:**
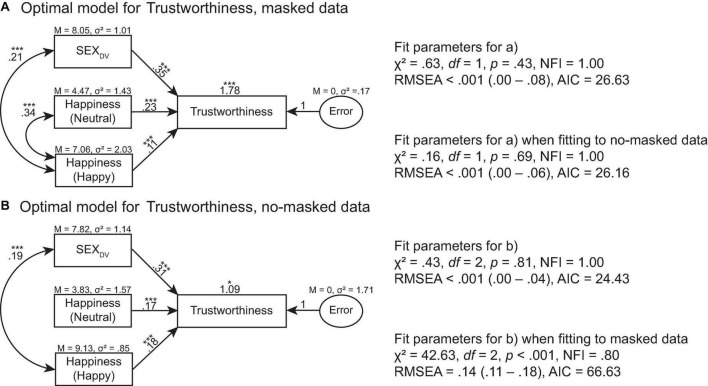
Optimal models of Trustworthiness for **(A)** masked face data and **(B)** no-masked face data. The optimal model was then fitted onto the alternate data to evaluate the fit estimates. For example, the optimal model for masked face data was fitted onto no-masked face data to determine if the optimal model for masked face data could predict no-masked face data. ***p > 0.001, **p > 0.01, *p > 0.05.

The optimal model of Trustworthiness for masked data was predicted by SEX_DV_, Happiness (Neutral), and Happiness (Happy) ratings, χ^2^ = 0.63, *df* = 1, *p* = 0.43, NFI = 1.00, RMSEA = 0.00 (0.00–0.08), AIC = 26.63 (Figure 6A). This model also predicted Trustworthiness for no-masked data, χ^2^ = 0.16, *df* = 1, *p* = 0.69, NFI = 1.00, RMSEA < 0.001 (0.00–0.06), AIC = 26.16. The optimal model of Trustworthiness for no-masked data was predicted by SEX_DV_, Happiness (Neutral), and Happiness (Happy) ratings, χ^2^ = 0.43, *df* = 2, *p* = 0.81, NFI = 1.00, RMSEA < 0.001 (0.00–0.04), AIC = 24.43 (Figure 6B). This model could not predict Trustworthiness for masked data, χ^2^ = 42.63, *df* = 2, *p* < 0.001, NFI = 0.80, RMSEA = 0.14 (0.11–0.18), AIC = 66.63.

#### Attractiveness

**FIGURE 7:**
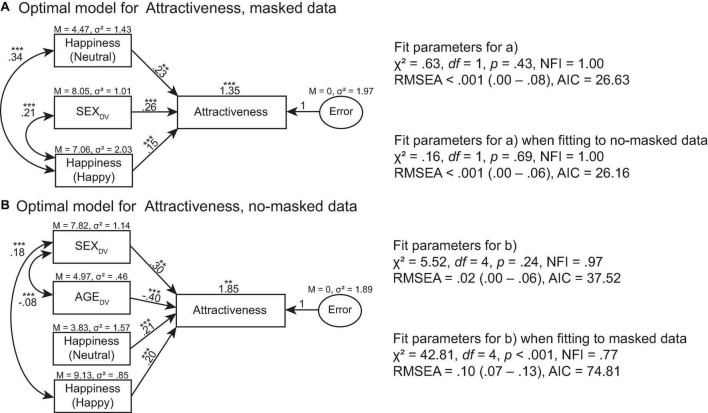
Optimal models of Attractiveness for **(A)** masked face data and **(B)** no-masked face data. ***p > 0.001, **p > 0.01, *p > 0.05.

The optimal model of Attractiveness for masked data was predicted by Happiness (Neutral), SEX_DV_, and Happiness (Happy) ratings, χ^2^ = 0.63, *df* = 1, *p* = 0.43, NFI = 1.00, RMSEA < 0.001 (0.00 –0.08), AIC = 26.63 (Figure 7A). This model also predicted Attractiveness ratings for no-masked data, χ^2^ = 0.16, *df* = 1, *p* = 0.69, NFI = 1.00, RMSEA < 0.001 (0.00–0.06), AIC = 26.16. The optimal model of Attractiveness for no-masked data was predicted by SEX_DV_, AGE_DV_, Happiness (Neutral), and Happiness (Happy) ratings, χ^2^ = 5.52, *df* = 4, *p* = 0.24, NFI = 0.97, RMSEA = 0.02 (0.00 –0.06), AIC = 37.52 (Figure 7B). This model did not predict Attractiveness ratings for masked data, χ^2^ = 42.81, *df* = 4, *p* < 0.001, NFI = 0.77, RMSEA = 0.10 (0.07–0.13), AIC = 74.81.

#### Approachability

**FIGURE 8:**
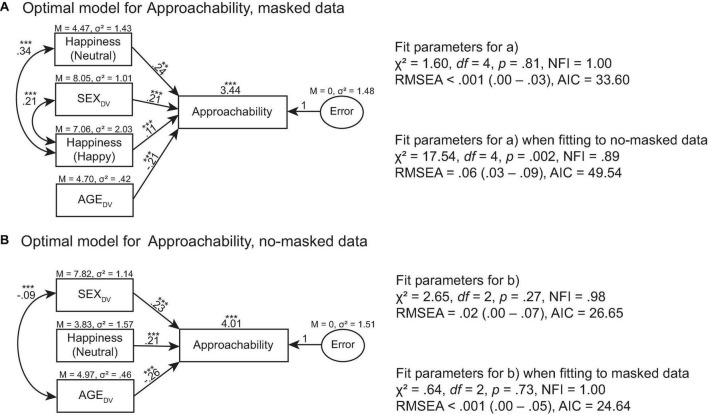
Optimal models of Attractiveness for **(A)** masked face data and **(B)** no-masked face data. ***p > 0.001, **p > 0.01, *p > 0.05.

The optimal model of Approachability for masked data was predicted by Happiness (Neutral), SEX_DV_, Happiness (Happy), and AGE_DV_ ratings, χ^2^ = 1.60, *df* = 4, *p* = 0.81, NFI = 0.99, RMSEA < 0.001 (0.00–0.03), AIC = 33.60 (Figure 8A). This model did not predict Approachability for no-masked data, χ^2^ = 17.54, *df* = 4, *p* = 0.002, NFI = 0.89, RMSEA = 0.06 (0.03–0.09), AIC = 49.54. The optimal model of Approachability for no-masked data was predicted by SEX_DV_, Happiness (Neutral), and AGE_DV_ ratings, χ^2^ = 2.65, *df* = 2, *p* = 0.27, NFI = 0.98, RMSEA = 0.02 (0.00–0.07), AIC = 26.65 (Figure 8B). This model also predicted Approachability for masked data, χ^2^ = 0.64, *df* = 2, *p* = 0.73, NFI = 1.00, RMSEA < 0.001 (0.00–0.05), AIC = 74.81.

These results suggested that, for some characteristics (i.e., Trustworthiness and Attractiveness), information retrieved from the visible portion of masked faces was also communicated when viewing full faces. In contrast, information retrieved when viewing the full face could not be used to understand partially occluded faces. Importantly, not all trait-like characteristics exhibited such findings (i.e., Approachability). For other trait-like characteristics, the information extracted when viewing full faces continued providing meaningful resources for discriminating partially occluded faces. However, information regarding these characteristics when viewing masked faces was not useful for full faces.

In conclusion, the results indicated that, for traits like trustworthiness and attractiveness, information from the unoccluded regions in masked faces could also be used to estimate the same trait for full faces. Likewise, for traits like approachability, information of a full face can maintain its utility in helping us understand the same trait in masked faces. Thus, the information from the unoccluded regions of the face used to evaluate a trait is dependent on the trait itself.

## Discussion

We investigated the role of the non-occluded regions of the face in communicating sex, age, attractiveness, trustworthiness, approachability, perceived excitability, and perceived happiness. Ratings for masked faces were significantly different from ratings for no-masked ones. We replicated the result that face masks confused happiness recognition. We also found that masked faces received favorable ratings when compared to no-masked faces. Participants were more accurate at judging the sex of masked faces. Masked faces also appeared younger, more trustworthy, more attractive, and more approachable. This evidence argued against the negative role of face masks in social interactions. Importantly, our results showed that the information extracted from masked faces remained valid in helping us understand the same information for no-masked faces. In some cases, information extracted from no-masked faces could aid our understanding of masked faces.

### The Impact of Face Masks on Social Interaction

Face masks occlude a gross part of the face which causes some facial expressions to be misinterpreted (Carbon, 2020). Such is an argument associating the negative impacts of face masks on social interactions. One method of showing such negative effects occurs when ratings for masked faces demonstrate central tendency or regression to the mean biases. This occurs because participants were unsure of the ratings or could not accurately estimate the characteristics (Tversky and Kahneman, 1974; Klopfer and Madden, 1980; Kulas and Stachowski, 2009). Ratings which deviated from a scale’s midpoint suggested that participants were certain of their judgments. From this rationale, our data provided some evidence about the negative impacts of face masks on emotion recognition. Specifically, the results replicated the literature showing that happiness was difficult to interpret for masked faces than no-masked faces (Carbon, 2020). This could be an evidence to argue that face masks negatively impacted social interaction in understanding happiness since the mouth region was a critical facial feature for communicating happiness (Beaudry et al., 2014). When crucial information from the mouth was blocked by the face masks, the ability to recognize happiness became severely impaired. However, we disagree with such conclusions. While face masks impede emotional expressions, it does not always impact social interactions negatively.

Face masks do not necessarily impact social interactions negatively. From our data, participants were better at identifying the sex of masked faces. Masked faces were also younger, more trustworthy, attractive, and approachable. If face masks only impacted social interactions negatively, then participants would experience more difficulty judging the sex. Similarly, masked faces should look older, less trustworthy, less attractive, or even less approachable, since these attributes would become less desirable in social interactions (Rotter, 1971; Perlini et al., 1999). Suffice to say, the conclusion that face masks negatively impact social interactions due to the impairments in emotional recognition should not be drawn hastily.

Social interaction in a dynamic and complex process. It also involves more than what can be perceived, or what that is lacking, from the mouth and mouth regions due to face masks. At the individual level, social interaction involves cognitive processes of both the self and the other (Doise et al., 1975). It also involves some level of awareness about others or their surroundings (Glaser and Strauss, 1964). Greater awareness often leads to higher acceptance, regardless of whether the awareness is about medical care (Hinton, 1999; Ozdag and Bal, 2001), consumerism (Gunes and Tekin, 2006), or interpersonal relationships (Krafft et al., 2017). Importantly, social interaction is also shaped by situational context (Rogoff and Lave, 1984; De Jaegher et al., 2010). The increased prevalence of wearing face masks, whether as an official directive, or by observing others with face masks, has likely led to a greater acceptance of face masks. Societal norms could further shape the general acceptance of face masks (Carbon, 2021). When exposed to such social contexts, it would not be surprising that more people now find the wearing of face masks acceptable and favorable as the act of putting on face masks becomes the norm.

The acceptance of face masks was exhibited in our data. We saw higher ratings for trustworthiness, attractiveness, and approachability for masked faces than no-masked ones. This result also supported recent findings investigating the link between face masks and the proximity between others (Cartaud et al., 2020). Specifically, participants reported higher willingness to be close to another person wearing face masks than to others without face masks. While one may argue that facial features or face symmetry can influence trait-like characteristics such as attractiveness (Perrett et al., 1999; Rhodes et al., 2000; Jones et al., 2007), the higher ratings for the trait-like characteristics in masked faces were unlikely explained by facial features alone. Roughly half the face was blocked by the face masks. A large portion of facial features was disturbed, making the evaluation of attractiveness purely by face symmetry difficult.

We speculate that a greater acceptance of face masks led to higher ratings in several characteristics of faces with face masks. For trustworthiness, some faces appear less trustworthy because of face proportions (Costa et al., 2017; Ormiston et al., 2017), posture (Okubo et al., 2018; Zhang et al., 2020), or facial expressions (Oosterhof and Todorov, 2009). However, the presence of face masks could modify the face‘s trustworthiness. Low-trustworthy faces were rated higher in trustworthiness when the face was covered by face masks as compared to the full face (Marini et al., 2021). For attractiveness, one study showed that masked faces appeared more attractive and healthier than full faces (Kamatani et al., 2021). Incidentally, masked faces could be approachable because they appear healthy looking. This could also partly explain why observers maintain close distances with face mask wearers (Cartaud et al., 2020; Calbi et al., 2021). Eventually, the oddity of wearing face masks decreases and people become more accustomed to them (Carbon, 2021). As face masks become more prevalent, the acceptance of the masks would increase, and face masks would be rated more favorable in social interactions.

### The Eyes and Eye Regions in Social Interaction

When the face is partially occluded by face masks, the unoccluded parts of the face (i.e., eyes and eye regions) continue to communicate information about the mask wearer because they remain visible. According to the literature, observers rely on the eyes to correctly recognize facial expressions when the mouth provides unreliable signals. The eyes also enhance the overall perceived happiness and trustworthiness of a face. The exception occurs only when the mouth is more reliable than the eyes in conveying signals about the face (Fernández-Martín et al., 2017). Our modeling data confirmed such findings (Figures 6–8). For example, trustworthiness model for masked faces predicted trustworthiness for no-masked faces. Since the difference between masked and no-masked faces was the occlusion, this signified that trustworthiness cues extracted from masked faces remained useful for evaluating trustworthiness in no-masked faces. Our models also exhibited such patterns for attractiveness ratings. Hence, we argued that the eyes and eye regions play an important role in social interaction, regardless of whether the face is fully visible or partially occluded.

There is evidence from the literature supporting the argument that the eyes and eye regions play an important role in social interaction. The eyes convey obvious social cues through facial expressions like fear, anger, and sadness (Dadds et al., 2006; Wegrzyn et al., 2017). One can also derive subtler characteristics like arousal and trustworthiness from the pupils (Bradshaw, 1967; Bradley et al., 2008; Kret et al., 2015; Wang et al., 2018). The eyebrows, also convey social signals about approachability or threat (Tipples et al., 2002). Based on these findings, it is imperative that one does not neglect the role of the eyes and eye region in social interactions. Thus, the eyes and eye regions also play important roles in social interactions.

### Future Directions

What we have learned from our data was that participants did not rate all characteristics negatively from the survey. We speculated that the greater acceptance of face masks could have translated into higher perceived characteristics for faces with face masks. This could be measured in future experiments by showing that higher ratings for the trait-like characteristics occur only for faces occluded by face masks, but not for faces occluded by other apparel. It would also be beneficial for future experiments to directly test if the ratings differed between static images and dynamic videos since social interaction is a complex and dynamic process. This would help generalize the results to real-world scenarios.

This study was limited by the unequal gender distribution of the sample. Since Calbi et al. (2021) found that face masks did not influence a female observer‘s ability to recognize emotions, the observer’s age and sex on perceived characteristics of a face should be investigated in future studies. It is also known that the age and sex of a face can influence face perception. For instance, females are better than males at evaluating faces (Herlitz and Lovén, 2013). Observers are also better at recognizing faces with similar age (Mason, 1986). Female faces are preferred over male faces (Cross and Cross, 1971; Penton-Voak et al., 2004) and the bias occur as early as during infancy (Quinn et al., 2002). Future studies could investigate the interaction between the impacts of face masks on the age and sex of the face.

What other information is conveyed by the non-occluded regions of faces with face masks? The literature hints that one needs only 100 ms when viewing novel faces to derive some characteristics about the person (Willis and Todorov, 2006; Todorov et al., 2009). In addition, where do the participants look when evaluating faces with face masks? Future research could investigate how the non-occluded regions of the face influence the perceived characteristics of the masked face, and employ eye tracking to measure where observers look when they evaluate masked faces.

## Conclusion

Face masks do not necessarily impact social interactions negatively. Although it can be more difficult to recognize some facial expressions over others, such evidence is not enough to conclude that face masks play a negative role in social interactions. Our data showed that emotional expressions were difficult to rate due to the face masks. However, other characteristics were favored for masked faces. Our modeling also showed that some information derived from viewing masked faces helped us understand no-masked faces, vice versa. We speculated that a greater acceptance of face masks could have driven the positive ratings for masked faces and offered some suggestions for future research directions.

## Data Availability Statement

The datasets presented in this study can be found in online repositories. The names of the repository/repositories and accession number(s) can be found below: https://oparu.uni-ulm.de/xmlui/handle/123456789/36828.

## Ethics Statement

Ethical review and approval was not required for the study on human participants in accordance with the local legislation and institutional requirements. The patients/participants provided their written informed consent to participate in this study.

## Author Contributions

The author confirms being the sole contributor of this work and has approved it for publication.

## Conflict of Interest

The author declares that the research was conducted in the absence of any commercial or financial relationships that could be construed as a potential conflict of interest.

## Publisher’s Note

All claims expressed in this article are solely those of the authors and do not necessarily represent those of their affiliated organizations, or those of the publisher, the editors and the reviewers. Any product that may be evaluated in this article, or claim that may be made by its manufacturer, is not guaranteed or endorsed by the publisher.
