# Supplementary material for: Face Masks Bolsters the Characteristics From Looking at a Face Even When Facial Expressions Are Impaired
Source: Front Psychol. 2021 Dec 10;12:704916. doi: 10.3389/fpsyg.2021.704916 (PMC8702500; doi:10.3389/fpsyg.2021.704916)
Supplement: Supplementary file 1 [file Data_Sheet_1.docx]

**Appendix**

Stepwise regression to derive optimal models

The role of the stepwise regression was to obtain optimal models of trait-like characteristics (Trustworthiness, Attractiveness, Approachability) predicted by invariant characteristics (SEX_DV_ & AGE_DV_) and emotional expressions (Excitability & Happiness). Each trait-like characteristic was estimated separately to avoid multicollinearity issues due to their correlation. The optimal model was decided using the following criteria: R^2^ change improved by only 0.1 after the predictor was introduced to the model. This criteria was utilized to keep the optimal model as parsimonious as possible.

The intercept for some models did not reached significance although the predictors contributed to data prediction. In such cases, we ran backwards regression to discard the most recent added predictors. This step was continued until the model produced a significant intercept. Our reason for including models with significant intercepts was that there should not be any biases when viewing a face. This assumption should be true in the absence of evaluating the invariant characteristics or emotional expressions of a face. The assumption, as translated into model form, was depicted by the significant intercept: when all predictors were set at zero, data would originate at the same starting point.

Stepwise models for masked face data

Each step in the stepwise regression for masked face data was detailed in Table 1. Trustworthiness was best predicted by SEX_DV_, Happiness (Neutral) and Happiness (Happy) as predictors. This model achieved an overall R^2^ = .14. From the model, Trustworthiness = .35 SEX_DV_ + .23 Happiness (Neutral) + .11 Happiness (Happy) + 1.78.

Attractiveness was best predicted by Happiness (Neutral), SEX_DV_, Happiness (Happy), AGE_DV_, and Happiness (Sad). The most parsimonious model for Attractiveness was one in which the R^2^ improved by only 0.1 after the predictor was introduced. Thus, the optimal model for Attractiveness contained three predictors. This model achieved an overall R^2^ = .11. From the model, Attractiveness = .23 Happiness (Neutral) + .26 SEX_DV_ + Happiness (Happy) + 1.35.

Approachability was best predicted by Happiness (Neutral), SEX_DV_, Happiness (Happy), AGE_DV_, Excitability (Happy), Excitability (Sad), and Happiness (Sad). The most parsimonious model for Approachability was one in which the R^2^ improved by only 0.1 after the predictor was introduced. Therefore, the optimal model for Approachability had four predictors. This model achieved an overall R^2^ = .12. From the model, Approachability = .24 Happiness (Neutral) + .21 SEX_DV_ + .11 Happiness (Happy) – .21 AGE_DV_ + 3.44.

| **Table 1.** Optimal models for predicting masked face data | | | | | |
| --- | --- | --- | --- | --- | --- |
| Trait-like characteristic | Predictors added | R^2^ | R^2^ change | F | *p* |
| Trustworthiness | SEX_DV_ | .08 | - | 79.55 | 2.24 × 10^-18^ |
|  | Happiness  (Neutral) | .12 | .04 | 70.00 | 3.72 × 10^-29^ |
|  | Happiness  (Happy) | .14 | .02 | 51.63 | 5.36 × 10^-31^ |
|  |  |  |  |  |  |
| Attractiveness | Happiness  (Neutral) | .05 | - | 50.08 | 2.80 × 10^-12^ |
|  | SEX_DV_ | .09 | .04 | 46.84 | 3.70 × 10^-20^ |
|  | Happiness  (Happy) | .11 | .01 | 39.11 | 7.01 × 10^-24^ |
|  | AGE_DV_ | .12 | .01 | 32.66 | 1.35 × 10^-25^ |
|  | Happiness  (Sad) | .13 | .01 | 28.06 | 1.24 × 10^-26^ |
|  |  |  |  |  |  |
| Approachability | Happiness  (Neutral) | .06 | - | 64.99 | 2.16 × 10^-15^ |
|  | SEX_DV_ | .09 | .03 | 50.11 | 1.88 × 10^-21^ |
|  | Happiness  (Happy) | .11 | .02 | 38.73 | 1.15 × 10^-23^ |
|  | AGE_DV_ | .12 | .01 | 32.61 | 1.48 × 10^-25^ |
|  | Excitability  (Happy) | .13 | .01 | 29.54 | 5.20 × 10^-28^ |
|  | Excitability  (Sad) | .14 | .01 | 25.69 | 1.87 × 10^-28^ |
|  | Happiness  (Sad) | .14 | - | 23.14 | 3.56 × 10^-29^ |
|  |  |  |  |  |  |
|  | Final model | Estimate | S.E. | *t* |  |
| Trustworthiness | Intercept | 1.78 | .38 | 4.65 | 4.00 × 10^-6^ |
|  | SEX_DV_ | .35 | .04 | 8.40 | 1.58 × 10^-16^ |
|  | Happiness (Neutral) | .23 | .04 | 6.63 | 5.41 × 10^-11^ |
|  | Happiness (Happy) | .11 | .03 | 3.63 | .0003 |
|  |  |  |  |  |  |
| Attractiveness | Intercept | 1.35 | .42 | 3.23 | .001 |
|  | Happiness (Neutral) | .23 | .04 | 6.03 | 2.32 × 10^-9^ |
|  | SEX_DV_ | .26 | .05 | 5.76 | 1.14 × 10^-8^ |
|  | Happiness (Happy) | .15 | .03 | 4.66 | 4.00 × 10^-6^ |
|  |  |  |  |  |  |
| Approachability | Intercept | 3.44 | .45 | 7.59 | 7.65 × 10^-14^ |
|  | Happiness (Neutral) | .24 | .03 | 7.20 | 1.23 × 10^-12^ |
|  | SEX_DV_ | .21 | .04 | 5.30 | 1.43 × 10^-7^ |
|  | Happiness (Happy) | .11 | .03 | 3.88 | .0001 |
|  | AGE_DV_ | -.21 | .06 | -3.58 | .0004 |
|  |  |  |  |  |  |

Stepwise models for no-masked face data

Each step in the stepwise regression for no-masked face data was detailed in Table 2. Trustworthiness was best predicted by SEX_DV_, Happiness (Neutral), Happiness (Happy), Happiness (Sad), and AGE_DV_. The most parsimonious model for Trustworthiness was one where the R^2^ improved by only 0.1 after the predictor was introduced. This corresponded to a model with four predictors. The model with four predictors was rejected because the intercept did not reach significance (*p* = .73). Therefore, the optimal model for Trustworthiness had three predictors, obtaining an R^2^ = .11. From the model, Trustworthiness = .31 SEX_DV_ + .17 Happiness (Neutral) + .18 Happiness (Happy) + 1.09.

Attractiveness was best predicted by SEX_DV_, AGE_DV_, Happiness (Neutral), Happiness (Happy), Happiness (Sad), and Excitability (Neutral). The parsimonious model for Attractiveness was one where the R^2^ improved by only 0.1 after a predictor was introduced. This corresponded to a model with four predictors and a model with six predictors. The model with six predictors was rejected because the intercept did not reach significance (*p* = .40). Therefore, the parsimonious model was one with four predictors, acheiving an R^2^ = .14. From the model, Attractiveness = .30 SEX_DV_ – .40 AGE_DV_ + .22 Happiness (Neutral) + .20 Happiness (Happy) + 1.85.

| **Table 2.** Optimal models for predicting no-masked faces | | | | | |
| --- | --- | --- | --- | --- | --- |
| Dependent variable | Predictors added | R^2^ | R^2^ change | F | *p* |
| Trustworthiness | SEX_DV_ | .07 | - | 74.90 | 1.96 × 10^-17^ |
|  | Happiness  (Neutral) | .09 | .02 | 50.95 | 8.72 × 10^-22^ |
|  | Happiness  (Happy) | .11 | .02 | 39.30 | 5.41 × 10^-24^ |
|  | Happiness  (Sad) | .12 | .01 | 32.57 | 1.58 × 10^-25^ |
|  | AGE_DV_ | .12 | - | 26.96 | 1.32 × 10^-25^ |
|  |  |  |  |  |  |
| Attractiveness | SEX_DV_ | .07 | - | 71.47 | 9.99 × 10^-17^ |
|  | AGE_DV_ | .10 | .03 | 53.39 | 9.61 × 10^-23^ |
|  | Happiness  (Neutral) | .13 | .03 | 48.91 | 1.79 × 10^-29^ |
|  | Happiness  (Happy) | .14 | .01 | 41.55 | 3.21 × 10^-32^ |
|  | Happiness  (Sad) | .16 | .02 | 37.29 | 4.46 × 10^-35^ |
|  | Excitability  (Neutral) | .17 | .01 | 32.42 | 9.73 × 10^-36^ |
|  |  |  |  |  |  |
| Approachability | SEX_DV_ | .04 | - | 45.03 | 3.27 × 10^-11^ |
|  | Happiness  (Neutral) | .08 | .04 | 41.40 | 5.41 × 10^-18^ |
|  | AGE_DV_ | .10 | .02 | 34.54 | 3.15 × 10^-21^ |
|  | Excitability  (Happy) | .10 | - | 28.11 | 3.94 × 10^-22^ |
|  | Happiness  (Happy) | .11 | .01 | 24.27 | 4.35 × 10^-23^ |
|  | Happiness  (Sad) | .12 | .01 | 22.95 | 1.94 × 10^-25^ |
|  | Excitability  (Neutral) | .13 | .01 | 20.58 | 6.40 × 10^-26^ |
|  |  |  |  |  |  |
|  | Final model | Estimate | S.E. | *t* |  |
| Trustworthiness | Intercept | 1.09 | .49 | 2.21 | .027 |
|  | SEX_DV_ | .31 | .04 | 7.86 | 9.77 × 10^-15^ |
|  | Happiness  (Neutral) | .17 | .03 | 5.12 | 3.77 × 10^-7^ |
|  | Happiness  (Happy) | .18 | .05 | 3.82 | .0001 |
|  |  |  |  |  |  |
| Attractiveness | Intercept | 1.85 | .64 | 2.92 | .004 |
|  | SEX_DV_ | .30 | .04 | 7.03 | 3.98 × 10^-12^ |
|  | AGE_DV_ | -.40 | .07 | -6.07 | 1.85 × 10^-9^ |
|  | Happiness  (Neutral) | .22 | .04 | 6.12 | 1.32 × 10^-9^ |
|  | Happiness  (Happy) | .20 | .05 | 4.13 | 3.90 × 10^-5^ |
|  |  |  |  |  |  |
| Approachability | Intercept | 4.01 | .45 | 8.99 | 1.22 × 10^-18^ |
|  | SEX_DV_ | .23 | .04 | 6.24 | 6.46 × 10^-10^ |
|  | Happiness (Neutral) | .20 | .03 | 6.28 | 4.99 × 10^-10^ |
|  | AGE_DV_ | -.26 | .06 | -4.39 | 1.30 × 10^-5^ |
|  |  |  |  |  |  |

Approachability was best predicted by SEX_DV_, Happiness (Neutral), AGE_DV_, Excitability (Happy), Happiness (Happy), Happiness (Sad), and Excitability (Happy). The most parsimonious model for Approachability was one in which the R^2^ improved by only 0.1 after the predictor was introduced. This corresponded to a model with three predictors, which obtained an R^2^ = .10. From the model, Approachability = .23 SEX_DV_ + .20 Happiness (Neutral) – .26 AGE_DV_ + 4.01.
